# Ethanolic Extracts of *Artemisia apiacea* Hance Improved Atopic Dermatitis-Like Skin Lesions In Vivo and Suppressed TNF-Alpha/IFN-Gamma–Induced Proinflammatory Chemokine Production In Vitro

**DOI:** 10.3390/nu10070806

**Published:** 2018-06-22

**Authors:** Ju-Hye Yang, Esther Lee, BoHyoung Lee, Won-Kyung Cho, Jin Yeul Ma, Kwang-Il Park

**Affiliations:** 1 Korean Medicine Application Center, Korea Institute of Oriental Medicine, 70 Cheomdan-ro, Dong-gu, Daegu 41062, Korea; jjuhye@kiom.re.kr (J.-H.Y.); lesd514@kiom.re.kr (E.L.); wkcho@kiom.re.kr (W.-K.C.); 2 ViroMed Co., Ltd., Seoul National University 1, Gwanak-ro, Gwanak-gu, Seoul 151-747, Korea; bhlee@viromed.co.kr

**Keywords:** *Artemisia apiacea* Hance, atopic dermatitis, keratinocytes, inflammation, chemokines

## Abstract

*Artemisia apiacea* Hance is a traditional herbal medicine used for treating eczema and jaundice in Eastern Asia including China, Korea, and Japan. However, the biological and pharmacological actions of *Artemisia apiacea* Hance in atopic dermatitis (AD) are not fully understood. An ethanolic extract of *Artemisia apiacea* Hance (EAH) was tested in vitro and in vivo to investigate its anti-inflammatory activity and anti-atopic dermatitis effects. The results showed that EAH dose-dependence inhibited production of regulated on activation, normal T-cell expressed and secreted (RANTES), interleukin (IL)-6, IL-8, and thymus and activation-regulated chemokine (TARC). EAH inhibited the activation of p38, extracellular signal-regulated kinases (ERK), and STAT-1 and suppressed the degradation of inhibited both nuclear factor of kappa light polypeptide gene enhancer in B-cells inhibitor-alpha (IκB-α) in TNF-α/IFN-γ–stimulated HaCaT cells. EAH also suppressed the translocation of inflammation transcription factors such as NF-κB p65 in TNF-α/IFN-γ–stimulated HaCaT cells. In addition, EAH reduced 2,4-dinitrochlorobenzene (DNCB)-induced ear thickness and dorsal skin thickness in a dose-dependent manner. EAH appeared to regulate chemokine formation by inhibiting activation of and ERK as well as the NK-κB pathways. Furthermore, EAH significantly improved the skin p38 conditions in a DNCB-induced AD-like mouse model.

## 1. Introduction

Atopic dermatitis (AD) is a chronic skin disease that affects millions of individuals worldwide [1]. The current drugs for AD such as corticosteroids may be effective but have serious side effects. Therefore, alternatives are urgently needed. Accordingly, alternative medicines such as herbal extracts with potent efficacy and minimal side effects have been considered in the development of AD treatments [2,3]. Current treatments to reduce inflammation and itching mainly involve the application of topical ointments and administration of oral medications [4].

Keratinocytes are the major cell types in the epidermis and maintain the biochemical and physical condition of the skin. They are also involved in the progression of various inflammatory skin diseases. Epidermal keratinocytes release inflammatory mediators such as pro-inflammatory cytokines and chemokines in response to immune triggers including ultraviolet light, allergens, and chemical agents [5]. Activated keratinocytes can produce interleukin (IL)-8 and IL-6, which mediate the influx of T cells and neutrophils into the epidermis. Thymus and activation-regulated chemokine (TARC/CCL17) and regulated normal T-cells expressed and secreted (RANTES), which are secreted from keratinocytes, have important roles in the infiltration of Th2 cells into inflammatory tissues [6,7,8,9,10]. To elucidate the anti-inflammatory effect of EAH, we activated HaCaT cells by treating them with tumor necrosis factor-α/interferon-γ (TNF-α/IFN-γ).

Repeated applications of dust mite extract and 2,4-dinitrochlorobenzene (DNCB) to mice have been reported to increase epidermal thickness in the DNCB-exposed dorsal skin [11]. These immunological changes are similar to those observed in AD patients [1]. We examined the anti-atopic efficacy of EAH in DNCB-induced atopic dermatitis models.

*Artemisia apiacea* is a traditional herbal medicine used in the treatment of eczema and jaundice in Eastern Asia including China, Korea, and Japan [12]. *A. apiacea* Hance is one of the most widely used herbs for treating malaria, jaundice, and dyspeptic complaints in oriental medicine [13]. In the early 1970s, artemisinin was isolated and identified as the active antimalarial ingredient. Thereafter, the effects of *A. apiacea* and artemisinin such as their anti-inflammatory, antipyresis, antibacterial, and antiparasitic effects as well as the immunosuppressive effects of *A. apiacea* extracts have been extensively studied. Recently, an *A. apiacea* extract was found to be effective against systemic anaphylactic shock [14] and to have an anti-inflammatory effect on lipopolysaccharide-activated Raw264.7 macrophage cells [13]. However, the biological and pharmacological actions of *Artemisia apiacea* Hance in atopic dermatitis (AD) are not completely understood.

In this study, we investigated the anti-inflammatory activity and the anti-atopic dermatitis effect of ethanolic extracts of *Artemisia apiacea* Hance (EAH) in vitro and in vivo.

## 2. Materials and Methods

### 2.1. Reagents

The ingredients of the complete cell culture medium, fetal bovine serum (FBS), and antibiotics were purchased from Lonza (Basel, Switzerland). Goat serum was purchased from Gibco (Grand Island, NY, USA). All other chemicals were made of a reagent grade. Quercetin-3-beta-d-glucoside and quercetin were purchased from Sigma-Aldrich (St. Louis, MO, USA). Scoparone was supplied by ChemFaces (Wuhan, Hubei, China). Trifluoroacetic acid was obtained from Sigma-Aldrich. High-performance liquid chromatography (HPLC)-grade acetonitrile was purchased from Merck (Merck KGaA, Darmstadt, Germany). HPLC-grade ultrapure water was obtained from a Puris-Evo UP Water system with an Evo-UP Dio VFT and an Evo-ROP Dico20 (Mirae ST Co., Ltd., Anyang, Korea). Ultrapure water was prepared with a resistivity of 18.2 MΩ cm^−1^ (Puris, Esse-UP Water System, Mirae St Co., Anyang, Korea). Recombinant human TNF-α, IFN-γ, and enzyme-linked immunosorbent assay (ELISA) kits for RANTES, IL-8, IL-6, and TARC were obtained from BioLegend (San Diego, CA, USA).

### 2.2. Culture of Keratinocytes

The human keratinocyte cell line HaCaT was provided by Dr. Misun Won, Korea Research Institute of Bioscience and Biotechnology. The cells were maintained in Dulbecco’s Modified Eagle’s medium with 10% FBS and penicillin/streptomycin under atmospheric conditions of 95% air and 5% CO_2_ at 37 °C. Cells in the third passage were used.

### 2.3. Preparation of Ethanolic Extract of Artemisia Apiacea Hance (EAH)

EAH was obtained from the Yeongcheon Oriental Herbal Market (Yeongcheon, Korea). All voucher specimens were deposited in the herbal bank of the KM-Based Herbal Drug Development Group, Korean Institute of Oriental Medicine (KIOM, Daegu, Korea) after being verified by Professor Ki-Hwan Bae of the College of Pharmacy, Chungnam National University (Daejeon, Korea). To prepare the EAH, dried *A. apiacea* Hance pieces (30.0 g) were extracted into 300 mL of 70% ethanol in a 40 °C shake-incubator for 24 h and filtered. Then 90 mL of 70% ethanol was added to the remaining residue, followed by shaking for 1 min, and then followed by filtration. The acquisition was 3.8623 g and the yield was 12.8743%. The freeze-dried extract powder was dissolved in dimethyl sulfoxide (DMSO) and centrifuged at 14,000 rpm (16,873 rcfs) for 10 min (Eppendorf™ Model 5418 Microcentrifuges; Epppendorf, Hamburg, Germany). The resulting supernatant was filtered (0.2 μm pore size) and then stored at 4 °C prior to use. In vitro experiments were performed by dissolving high concentrations of stock in 50% DMSO and a final concentration of DMSO <0.05% (non-toxic concentration of DMSO).

### 2.4. Chromatographic Conditions and Sample Preparation

The dried form of EAH was accurately weighed (25 mg) and dissolved in 1 mL of 100% methanol and then it was extracted by using ultra sonication for 30 min. The standard compound stock solutions were precisely weighed and dissolved in 100% methanol (1000 μg/mL). All working solutions (330 μg/mL, each) were filtered through 0.2 mm syringe membrane filters from Whatman Ltd. (Maidstone, UK) before being injected into the HPLC analysis system.

HPLC-UV/VIS diode array detector (DAD) studies were performed by using a Dionex Liquid Chromatography system (Dionex Co., Sunnyvale, CA, USA) fitted with an Ultimate 3000 Series binary pump, auto-sampler, column oven, and DAD. Chromatographic separation was achieved on a Waters Bridge C_18_ column (Waters Corporation, Milford, MA, USA). The column temperature was 40 °C, injection volume was 10 μL, and the flow rate was 1 mL/min for the total run time. The mobile phase comprised 0.1% trifluoroacetic acid in water (*v*/*v*) (A) and acetonitrile (B) [12]. The HPLC gradient was 10% to 60% B over 0–70 min. The detection wavelength was 254 nm. Data acquisition was performed by using the Dionex Chromelon (Dionex Co., Sunnyvale, CA, USA).

### 2.5. MTT Assay

Cell viability was determined by using a 3-(4,5-dimethylthiazol-2yl)-2,5-diphenyl-2*H*-tetrazolium bromide (MTT) agent. HaCaT cells (1 × 10^4^ cells/well) were seeded into 96-well plates and an EAH extract was added to the wells at concentrations ranging between 1 μg/mL and 200 μg/mL. After 24 h, MTT solutions were then added to each well and the cells were incubated for an additional 2 h. The resulting formazan was dissolved by DMSO and the optical density at 570 nm was determined by using an ELISA reader (Infinite M200; Tecan, Männedorf, Switzerland).

### 2.6. Cytokine and Chemokine Analysis

The cells (1 × 10^6^ cells/well) in 6 well plates were pretreated with EAH at indicated concentrations (1 μg/mL, 10 μg/mL, 50 μg/mL, and 100 μg/mL) for 1 h and stimulated with TNF-α/IFN-γ (each 10 ng/mL) for 24 h at 37 °C in a 5% CO_2_ atmosphere. After stimulation, the culture medium was then harvested and the levels of chemokines such as RANTES, IL-8, IL-6, and TARC were measured using ELISA kits, according to the manufacturer’s instructions. Recombinant human TNF-α, IFN-γ, and enzyme-linked immunosorbent assay (ELISA) kits for RANTES, IL-8, IL-6, and TARC were obtained from the BioLegend (San Diego, CA, USA). The plates were read at 450 nm and an inhibitory effect of EAH was determined from a standard curve.

### 2.7. Western Blot

Protein expression was evaluated by Western blot analysis according to standard procedures. The cells (1 × 10^6^ cells/well) in 6 well plates were pretreated with EAH at various concentrations (1 μg/mL, 10 μg/mL, 50 μg/mL, and 100 μg/mL) for 1 h and stimulated with TNF-α/IFN-γ (each 10 ng/mL) for 30 min at 37 °C in a 5% CO_2_ atmosphere. The primary antibodies (p38; p-p38 (Thr180/Tyr182); ERK1/2; p-ERK1/2 (Thr202/Tyr204); JNK; p-JNK (Thr183/Tyr185); STAT-1; p-STSA-1(Tyr701); IκBα; β-actin) and secondary antibodies (anti-rabbit (#7074); anti-mouse (#7076)) were obtained from Cell Signaling Technology (Boston, MA, USA). The Western blot was performed according to a previously described method [10].

### 2.8. Immunofluorescence Assay

The cells were seeded into a confocal dishes, incubated without or with EAH for 1 h, and stimulated with TNF-α/IFN-γ (each 10 ng/mL) for 20 min [15]. After treatment, cells were fixed with 4% paraformaldehyde in PBS. Fixed cells were blocked using 3% goat serum for 1 h at room temperature. After an overnight incubation at 4 °C with the anti-p65 and anti-STAT-1 antibodies at 4 °C, the cells were incubated with secondary Alexa-Fluor-488 and 568-labeled antibodies for 2 h at room temperature. The nuclei were stained with a nuclear marker, 4′,6-diamidino-2-phenylindole (DAPI; Sigma-Aldrich, St. Louis, MO, USA), for 10 min at room temperature. All samples were then observed using a confocal microscope (FV3000 FLUOVIEW; Olympus, Tokyo, Japan).

### 2.9. Animals

Male BALB/c mice (5 weeks old) were purchased from Samtako BioKorea (Osan, Korea). The mice were observed every day for 1 week during quarantine and acclimation. The mice were divided into five groups (*n* = 5 per group): (1) negative control (vehicle); (2) DNCB + vehicle (control); (3) DNCB + 50 mg/kg EAH; (4) DNCB + 100 mg/kg EAH; and (5) 1 mg/kg dexamethasone. All groups were maintained under standard conditions of temperature (22.5 ± 0.5 °C), humidity (42.6 ± 1.7%), 12 h lighting (8:00 a.m.–8:00 p.m., 290 lux), ventilation (10–15 times per hour), and diet (Teklad Global Diets, Harlan Laboratories Inc., ‎Indianapolis, IN,‎ USA). This study was conducted according to the guidelines listed in the Pharmaceutical Affairs Act of the Korea Food and Drug Association and approved by the Institutional Animal Care and Use Committee of Korea Conformity Laboratories (IA11-00920).

### 2.10. Induction of AD and Drug Treatment

The AD-like mouse model involved induction, which was described previously [16]. DNCB dissolved in an acetone:olive oil mixture (3:1 *v*/*v*), which was applied to the dorsal skin and both ears of BALB/c mice to induce AD-like symptoms and skin lesions. Test drugs were administered before applying DNCD. EAH dissolved in saline (10 mL/kg body weight) was orally administered by gavage 50 mg/kg or 100 mg/kg 3 times a week for 4 weeks (days 0–24) and dexamethasone (Sigma-Aldrich, 1 mg/kg) was used as a control drug.

### 2.11. Ear Thickness Measurements and Histopathological Analysis

For each mouse, ear thickness was measured and recorded with a micrometer (Mitutoyo, Kawasaki, Japan). To minimize variation, a single investigator performed all measurements. The dorsal skin lesions of each mouse were removed and fixed with 4% paraformaldehyde overnight at room temperature. For hematoxylin and eosin staining, fixed samples were analyzed by a commercial services company (Garam Meditech, Garam, South Korea). All samples were observed using a microscope and data were representative of five observations (Nikon Eclipse Ti; Nikon, Tokyo, Japan).

### 2.12. Statistical Analysis

The data were analyzed by using the GraphPad Prism version 5.0 (GraphPad Software, San Diego, CA, USA). The results are expressed as the means ± SEM and SD, which were evaluated by using Student’s *t*-test or the analysis of variance (one-way ANOVA, Dunnett, to compare control groups). A *p*-value of < 0.05 was considered for indicating statistical significance.

## 3. Results

### 3.1. Measurement of the Representative Component in EAH by HPLC Analysis

**Figure 1:**
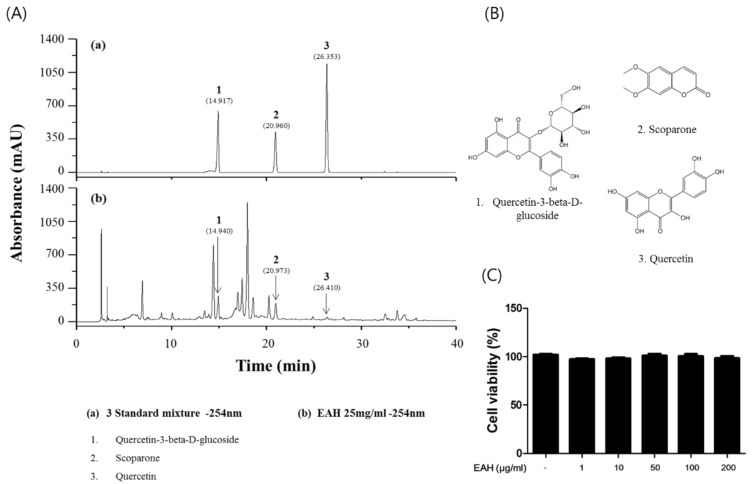
Measurement of representative components of extracts of Artemisia apiacea Hance (EAH) by HPLC analysis and cytotoxicity in HaCaT cells. (**A**) HPLC-DAD analysis chromatogram of three standard compound mixtures (**a**) and extract of *Artemisia apiacea* Hance (EAH) (**b**) that were identified at a wavelength of 254 nm. (**B**) Structures of the standard compounds. (**C**) Cell viability determined by 3-(4,5-dimethylthiazol-2yl)-2,5-diphenyl-2H-tetrazolium bromide (MTT) assay. The data are presented as the means ± SD of three experiments.

The three standard compounds of *A. apiacea* Hance were determined by HPLC. The wavelength that provided the maximum UV absorption of each compound was determined by using a DAD coupled to the HPLC system. The retention times and UV spectra of the peaks were compared with those of the respective standard compounds [17]. As shown in Figure 1A, the retention times of quercetin-3-beta-d-glucoside, scoparone, and quercetin in the standard solution were 14.917 min, 20.960 min, and 26.353 min, respectively. In the samples, the retention time of peaks 1, 2, and 3 were 14.940 min, 20.973 min, and 26.410 min, respectively, which closely matched those of the standard compounds (Figure 1B).

### 3.2. Effects of EAH on HaCaT Cell Viability

EAH was tested for cytotoxicity in human keratinocyte HaCaT cells by exposing them to various concentrations for 24 h. Figure 1C showed no toxic effects of EAH on cell viability up to a concentration of 200 µg/mL. Therefore, the cells were treated with doses lower than 200 µg/mL in subsequent experiments.

### 3.3. Effects of EAH on TNF-α/IFN-γ–Induced Production of Proinflammatory Cytokines and Chemokines in HaCaT Cells

**Figure 2:**
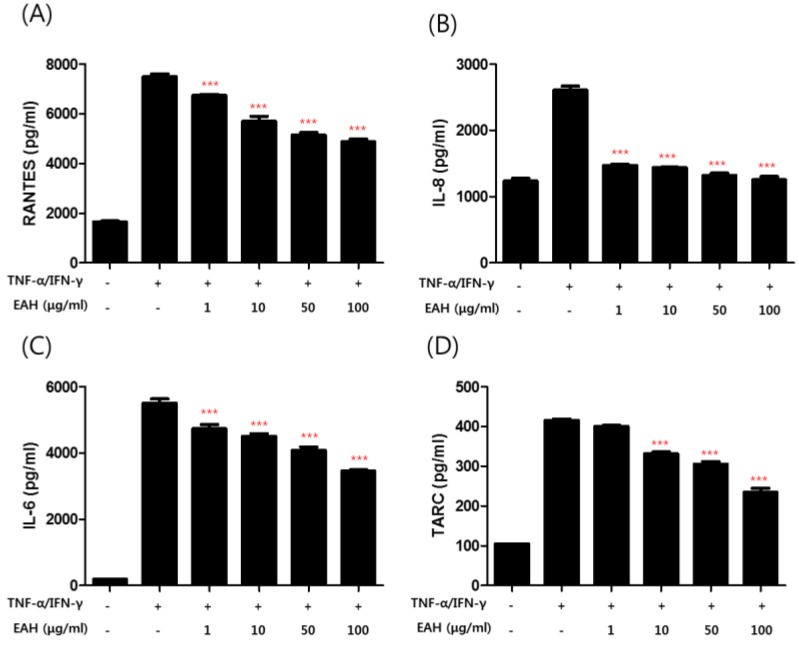
Effects of EAH on the TNF-α/IFN-γ–induced production of chemokines in HaCaT cells. The productions of RANTES (**A**), IL-8 (**B**), IL-6 (**C**), and TARC (**D**) were measured by using the culture supernatant of TNF-α/IFN-γ–stimulated HaCaT cells. The cells were pretreated with the indicated concentrations of EAH for 1 h and then stimulated with TNF-α/IFN-γ (each 10 ng/mL) for 24 h. Data are represented as mean ± SD of three independent experiments. *** *p* < 0.001 compared with the TNF-α/IFN-γ-treated group.

To investigate the anti-AD effect of EAH on pro-inflammatory cytokines and chemokines production upon TNF-α/IFN-γ co-stimulation, HaCaT cells were pretreated with EAH for 1 h followed by TNF-α/IFN-γ for 24 h and the supernatant was collected for cytokine level measurement by ELISA (Figure 2B,D). The results showed that EAH inhibited the production of RANTES, IL-6, IL-8, and TARC.

### 3.4. Effects of EAH on Phosphorylation of Mitogen Activated Protein Kinases (MAPK), STAT-1, and NFκB-p65 in TNF-α/IFN-γ–Stimulated HaCaT Cells

**Figure 3:**
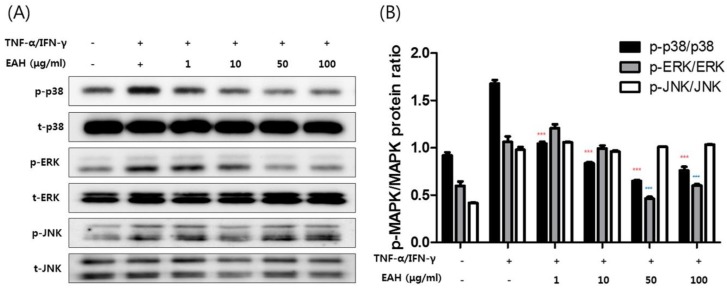

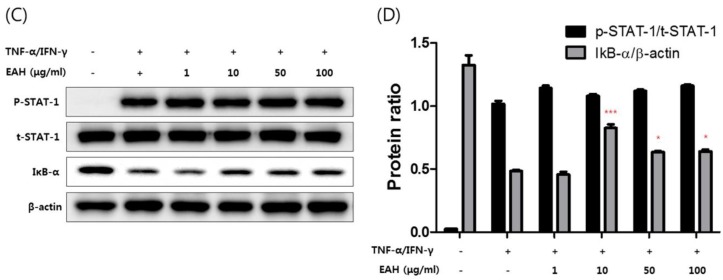
Effects of EAH on phosphorylation of MAPK and STAT-1 and degradation of IκB-α in TNF-α/IFN-γ–stimulated HaCaT cells. The cells were pretreated with the indicated concentrations of EAH for 1 h and then stimulated with TNF-α/IFN-γ (each 10 ng/mL) for 30 min. (**A**) Effects of EAH on phosphorylation of MAPKs such as P38, ERK, and JNK in HaCaT cells. (**B**) Relative abundances of proteins were calculated for the indicated protein ratios. (**C**) Effects of EAH on phosphorylation of STAT-1 and degradation of IκB-α in HaCaT cells. (**D**) The bar graphs represent quantitative densities of the bands. The results shown are representative of (**A**,**C**). Data are represented as mean ± SD of three independent experiments. * *p* < 0.05, *** *p* < 0.001, and ^###^ *p* < 0.001 compared with the TNF-α/IFN-γ-treated group.

We investigated the effects of EAH on the expression of inflammation-related factors such as MAPK, NFκB-p65, and STAT-1 in TNF-α/IFN-γ–stimulated HaCaT cells. Figure 3A shows the effects of EAH at 1–100 μg/mL on MAPK activities such as p38, extracellular signal-regulated kinases (ERK), and c-Jun N-terminal kinases (JNK). The relative abundances of proteins were calculated for the p-ERK/ERK, p-p38/p38, and p-JNK/JNK ratios (Figure 3B). As a result, EAH strongly inhibited the activation of p38 and ERK induced by TNF-α/IFN-γ without affecting the total protein level. In addition, we determined whether EAH affects transcription factors such as NFκB-p65 and STAT-1 in HaCaT cells. To investigate the effect of EAH on the NF-κB and STAT-1 signaling pathway, both the degree of IκB-α degradation and phosphorylation of STAT-1 were evaluated (Figure 3C). As shown in Figure 3C,D, treatment with EAH inhibited degradation of the nuclear factor-kappa B (NF-κB) inhibitory protein IκBα but does not inhibit phosphorylation of STAT-1.

### 3.5. Effects of EAH on NFκB-p65 and STAT-1 Translocation in TNF-α/IFN-γ-Stimulated HaCaT Cells

**Figure 4:**
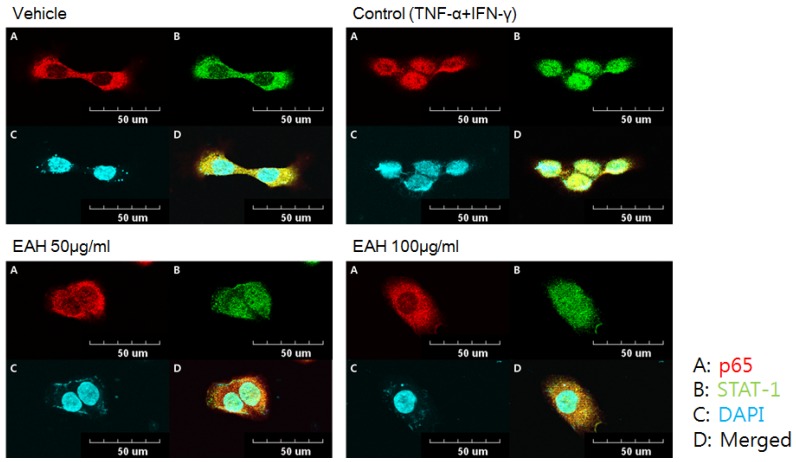
Effects of EAH on translocation of STAT-1 and NF-κB in TNF-α/IFN-γ–stimulated HaCaT cells. Confocal microscopy analysis of NF-κB p65 (A) and STAT-1 (B) in HaCaT cells. The cells were pretreated with EAH for 1 h and stimulated with TNF-α/IFN-γ for 20 min. Incubation was stopped and the cells were fixed in 4% formaldehyde and stained with the indicated primary antibody. HaCaTs were triple stained for NF-κB p65 (red, A), STAT-1 (green, B), and DAPI (blue, S). Merged images (D) indicate co-localization of STAT-1, NF-κB p65, and nucleus. Vehicle (DMSO), Control (vehicle + TNF-α/IFN-γ).

NFκB-p65 and STAT-1 are transcription factors that are critically involved in atopic dermatitis-related signaling in TNF-α/IFN-γ–stimulated HaCaT cells. Consequently, we confirmed the effect of EAH on nuclear translocation of NF-κB and STAT-1 in TNF-α/IFN-γ–stimulated HaCaT cells. Similar to Figure 3C results, treatment with EAH inhibited translocation of p65 from the cytoplasm to the nucleus but does not inhibit STAT-1 (Figure 4).

### 3.6. Effects of EAH on Development of DNCB-Induced AD Mouse Skin Lesions

**Figure 5:**
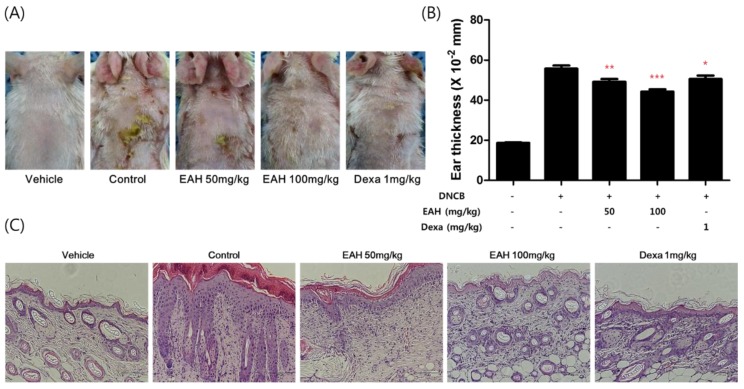
Effects of EAH on development of DNCB-induced AD mouse skin lesions. (**A**) Effects of EAH on clinical features of DNCB-induced AD skin lesions. (**B**) Ear thickness was measured by using a dial thickness gauge. (**C**) H&E stained DNCB-induced dorsal skin lesions (20×, scale bar = 100 µm). The results shown are representative of (**A**,**C**). Vehicle, acetone:olive oil mixture (3:1 vol/vol), Control, DNCB + Vehicle, EAH, DNCB + EAH treated group (50, 100 mg/kg), Dexa, DNCB + 1 mg/kg dexamethasone treated group. Data are represented as mean ± SEM of three independent experiments. * *p* < 0.05, ** *p* < 0.01, *** *p* < 0.001 compared with the control group.

Repeated cutaneous application of DNCB induces AD-like dermatitis in BALB/c mice. We investigated EAH following induction using DNCB of AD-like mouse skin lesions. As shown in Figure 5A, the skin conditions significantly prevented the EAH-administered groups relative to those in the control group. In addition, EAH prevented DNCB-induced ear thickness and dorsal skin thickness in a dose-dependent manner.

## 4. Discussion

AD is a chronic allergic inflammatory skin disease characterized by pruritic eczema and scratching behavior that can induce skin injury. Patients with AD exhibit elevated serum IgE levels and markedly increased levels of inflammatory cells including eosinophils, mast cells, and lymphocytes. Various cells involved in the allergic reaction infiltrate the lesions. Among the infiltrating cells, T helper 2 (Th2) cells are one of the most important cell types involved in AD development [18]. Although Th2 cells are the principal type of cells involved in the acute reaction of AD and Th1 cells are highly expressed during the chronic AD phase. Until now, local or systemic glucocorticosteroids as well as emollients are the main therapeutic modalities for control of AD [19], but their long-term use is controversial because these steroids can produce side effects in AD patients. Therefore, many researchers are trying to develop more effective and safer treatments for AD.

HaCaT cells are one of the cell lines used to mimic AD symptoms in response to inflammatory stimuli such as TNF-α/IFN-γ [20]. Stimulation of keratinocytes by TNF-α/IFN-γ leads to activation of various signaling pathways that involve STAT-1, NF-κB, and MAPKs, which subsequently increase expressions of inflammatory mediators [21]. In this study, we activated HaCaT cells by treating them with TNF-α/IFN-γ for investigating the anti-inflammatory effect of EAH. The results showed that EAH inhibited the production of chemokines and pro-inflammatory cytokines such as RANTES, IL-8, IL-6, and TARC (Figure 2). We also investigated how EAH inhibits the secretion of inflammatory cytokines and chemokines. As a result, EAH inhibited the activation of p38, ERK without JNK inhibition.

NF-κB and STAT-1 are protein transcription factors that are required for the transcription of a wide array of pro-inflammatory molecules in AD. In the resting state, NF-κB dimers are inactive in the cytoplasm of cells and are associated with the IκB. Upon stimulation with TNF-α/IFN-γ, the IκB-kinase complex is activated and phosphorylates IκB, which leads to the substrate’s ubiquitination and subsequent degradation. The resulting free NF-κB is translocated to the nucleus where it can activate target genes by binding to regulatory elements in the target gene’s promoter. Similarly, STAT-1 is phosphorylated and activated when stimulated with TNF-α/IFN-γ and translocated to the nucleus where it can activate target genes by binding to regulatory elements in the target gene’s promoter [22]. As shown in Figure 3, EAH inhibited degradation of the nuclear factor-kappa B (NF-κB) inhibitory protein IκBα but did not inhibit phosphorylation of STAT-1. Furthermore, we observed the effect of EAH on transcription factors such as NF-κB and STAT-1. We confirmed that EAH inhibited the translocation of NF-κB p65.

The ability of TNF-α/IFN-γ to synergistically induce production of cytokines and chemokines by keratinocytes has been exploited in an inflammatory skin disease. The pro-inflammatory cytokines and chemokines such as IL-6, IL-8, TARC, and RANTES have pivotal roles in progressing inflammation as a result of monocyte activation [1,23]. These common mediators activate and recruit immune cells to chronic inflammatory sites and the infiltration of Th2-type lymphocytes into skin lesions is associated with high expression of cytokines and chemokines in AD patients [6,7,8,9,10]. Moreover, TNF-α/IFN-γ attenuates activation of the STAT-1/Jak-2 and NK-κB pathways [24,25,26]. Many researchers have reported that TNF-α/IFN-γ induces the production of pro-inflammatory chemokines and cytokines such as RANTES, TARC, IL-6, and IL-8 and activates MAPK via JAK/STAT or NK-κB pathways in human epidermal keratinocytes [26,27,28,29].

Recently, many types of mouse models similar to our AD-like model have been developed. In one model, the ears or dorsal skin of a BALB/c mouse is repeatedly exposed to chemicals DNCB, trinitrochloridebenzene, and 1-fluoro-2,4-dinitrobenzene. Another model is the NC/Nga mice that have developed AD-like symptoms under conventional but not specific pathogen-free conditions [30]. Repeated cutaneous application of DNCB induces AD-like dermatitis in BALB/c mice. In AD-like skin by DNCB, many changes such as a thick epidermis of the epidermis and dermis, significant keratosis, infiltration of inflammatory cells, ulceration, and hemorrhage are observed [31]. We found that EAH restrained edema of the ear skin and restored epidermal thickness through the histological analysis (Figure 5).

In this study, we demonstrated the mechanism underlying the anti-inflammatory activity of EAH in HaCaT cells and investigated the anti-AD effects in an AD-like mouse model. We found that EAH regulated expression of pro-inflammatory cytokines and chemokines via the p38/NF-κB pathway in allergic inflammation. In addition, EAH inhibited the degradation of IκBα and the translocation of NF-κB p65. Moreover, EAH improved the skin lesions and reduced the thickness of dorsal skin and ear skin thickness in a DNCB-induced AD-like model. These results indicate that EAH can therapeutically reduce AD-like symptoms in mice. Our results suggest that EAH is a promising adjuvant treatment for AD. However, many questions remain to be addressed and additional studies such as serum IgE, cytokine, chemokine levels, and histological analysis are needed.
